# Association between time of discharge from ICU and hospital mortality: a systematic review and meta-analysis

**DOI:** 10.1186/s13054-016-1569-x

**Published:** 2016-12-01

**Authors:** Si Yang, Zheng Wang, Zhida Liu, Jinlai Wang, Lijun Ma

**Affiliations:** Department of Respiratory and Critical Care Medicine, Zhengzhou University People’s Hospital, Zhengzhou, Henan China

**Keywords:** Hospital mortality, Critically ill, ICU, Discharge, Weekend, Nighttime, Systematic review, Meta-analysis

## Abstract

**Background:**

Epidemiological studies have provided inconsistent results on whether intensive care unit (ICU) discharge at night and on weekends is associated with an increased risk of mortality. This systematic review and meta-analysis aimed to determine whether ICU discharge time was associated with hospital mortality.

**Methods:**

The PubMed, Embase, and Scopus databases were searched to identify cohort studies that investigated the effects of discharge from the ICU on weekends and at night on hospital mortality, with adjustments for the disease severity at ICU admission or discharge. The primary meta-analysis focused on the association between nighttime ICU discharge and hospital mortality. The secondary meta-analysis examined the association between weekend ICU discharge and hospital mortality. Odds ratios (ORs) with 95% confidence intervals (CIs) were pooled using a random-effects model.

**Results:**

We included 14 studies that assessed outcomes for nighttime versus daytime discharges among 953,312 individuals. Of these 14 studies, 5 evaluated outcomes for weekend versus weekday discharges (n = 70,883). The adjusted OR for hospital mortality was significantly higher among patients discharged during the nighttime, compared to patients discharged during the daytime (OR 1.31, 95% CI 1.25–1.38, *P* < 0.0001), and the studies exhibited low heterogeneity (*I* ^2^ = 33.8%, *P* = 0.105). There was no significant difference in the adjusted ORs for hospital mortality between patients discharged during the weekend or on weekdays (OR 1.03, 95% CI 0.88–1.21, *P* = 0.68), although there was significant heterogeneity between the studies in the weekday/weekend analysis (*I* ^2^ = 72.5%, *P* = 0.006).

**Conclusions:**

Nighttime ICU discharge is associated with an increased risk of hospital mortality, while weekend ICU discharge is not. Given the methodological limitations and heterogeneity among the included studies, these conclusions should be interpreted with caution, and should be tested in further studies.

**Electronic supplementary material:**

The online version of this article (doi:10.1186/s13054-016-1569-x) contains supplementary material, which is available to authorized users.

## Background

The in-hospital mortality rate among intensive care patients is 20–30%, and these patients account for 20–50% of all hospital deaths [1, 2]. Thus, clinicians, hospital administrators, and policy makers are challenged to reduce hospital mortality among critically ill patients. The greatest risk of death is related to the intensive care unit (ICU) admission, triage, and discharge, with up to 10.8% of patients dying after being discharged from the ICU [3, 4]. In this context, death after ICU discharge is predicted by a higher acute physiology score, organ or system failure, older age, prolonged hospitalization, discharge destination, and a do-not-resuscitate order [3, 4].

It is only in recent years that the possible relationship between time of discharge from ICU and hospital mortality has been recognized [5]. Nighttime ICU discharge refers to discharge from ICU at night and during out-of-office hours, and is also known as an “out-of-hours discharge”, “after-hours discharge”, “nighttime transfer”, or “night shift transfer”. Retrospective and prospective studies from the UK [6], Australia [7–10], Canada [11, 12], and the USA [13] have highlighted the risks of adverse outcomes that may be associated with nighttime ICU discharge. These unfavorable outcomes may include greater in-hospital mortality [6, 10], a higher unplanned ICU readmission rate [9, 13], and prolonged hospitalization [13]. However, several other studies, including the most recent large-scale prospective study, failed to draw similar conclusions [14, 15], and similar inconsistencies have been observed in studies of whether weekend discharge is harmful [8, 10, 12, 14, 16].

These discrepancies are likely influenced by the local healthcare systems, patient populations, definitions of nighttime or weekend discharge, disease severity at admission or discharge, therapy limitations, sample size, and study design. Nevertheless, no study has comprehensively examined the discrepancies and similarities among these research results.

Evidence-based practical guidelines have cited the existing research data to support their suggestions on ICU discharge time [17, 18]. Unfortunately, the strength of these recommendations is relatively weak. For example, the UK Faculty of Intensive Care Medicine and the Intensive Care Society suggest avoiding nighttime discharge (between 22:00 and 06:59) to reduce mortality and patient discomfort [17], although this suggestion cites only two retrospective studies [11, 12]. Based on a more comprehensive literature search, the newly revised American Society of Critical Care Medicine ICU practice guidelines recommend avoiding nighttime ICU discharge but not weekend discharge [18]. However, these two practical recommendations were graded as evidence level 2C (the highest evidence level is 1A), because they were formulated using a consensus review of contradictory research evidence [18].

Thus, there is a need for stronger evidence that combines all relevant data in a quantitative manner. We performed this systematic review and meta-analysis to identify whether nighttime or weekend ICU discharge is associated with hospital mortality.

## Methods

### Data sources and search strategy

Two independent investigators (SY and JW) performed a systematic search, without language or publication type restrictions, of the PubMed, Embase, and Scopus databases from their inception to 1 August 2016. The searches used a combination of the following search terms with the appropriate wildcards and spelling variations: “intensive care unit”, “night-shift”, “night”, “nighttime”, “out-of-hours”, “evening”, “off-hour”, “after-hours”, “time”, “discharge”, “transfer”, “mortality”, and “death”.

The search was limited to studies of human adults (Additional file 1: Table S1). Publications in non-English languages (e.g., French, Japanese, or German) were translated by an independent translation service. Additional searches were performed using two clinical trial registries (http://clinicaltrials.gov/ and http://www.isrctn.com/), and abstracts from major international conferences were manually searched at their official journal websites (Society of Critical Care Medicine: Critical Care Medicine (1998–2015); American Thoracic Society: American Journal of Respiratory and Critical Care Medicine (2009–2016); European Society of Intensive Care Medicine: Intensive Care Medicine (1988–2014); International Symposium on Intensive Care and Emergency Medicine: Critical Care (1997–2016); American College of Chest Physicians: Chest (2003–2015); Australian and New Zealand Intensive Care Society Annual Meeting: Anaesthesia and Intensive Care (1990–2015)).

Articles that were published online ahead of print in major intensive care journals were searched manually. The two investigators also reviewed the reference lists (Additional file 1: Table S1) of the retrieved studies and relevant reviews to identify additional articles [19, 20]. In instances where further clarification was required, a third investigator (ZW) emailed the corresponding author of the relevant article.

The meta-analysis was pre-specified and performed according to the Preferred Reporting Items for Systematic Reviews and Meta-Analyses (PRISMA) criteria (Additional file 2) [21]. The meta-analysis and systematic review protocol has not been published, and is not registered with the PROSPERO database or the Cochrane Library.

### Study selection

Studies were considered eligible if they fulfilled the following criteria: (1) a cohort study design; (2) a study population of mainly adult patients who were discharged alive from an ICU (general surgical, medical, or mixed) and were grouped into nighttime/daytime discharges and/or weekday/weekend discharges, and the study assessed outcomes for nighttime versus daytime discharges or outcomes for weekend versus weekdays discharges; (3) the primary outcome was hospital mortality among the patients who were discharged from the ICU (according to their nighttime, daytime, weekday, and/or weekend grouping); and (4) the study reported the effect size and 95% confident interval (CI) with adjustment for disease severity (or data to calculate these results).

Studies were excluded if they fulfilled any of the following criteria: (1) the study population comprised mainly pediatric patients; (2) the study population comprised patients discharged from a high-dependency or step-down unit; (3) there was no control population; (4) the study was not original research; or (5) the study did not provide sufficient information for data extraction and quality assessment (even after contacting the relevant authors). In cases of duplicate publication, we only included the most informative and complete study (typically the most recent publication).

Two investigators (SY and JW) independently screened the titles and abstracts of all citations. The full-text articles were retrieved for full-text review if either investigator thought that the citation might fulfill our eligibility criteria. The same two investigators independently evaluated the eligibility of all full-text articles that were selected during the screening process, and the κ value (i.e., chance-independent agreement) was found to be 0.82. Disagreements were resolved through a consensus process in which investigators discussed the reasoning behind their decisions. In all disagreements, one of the investigators realized that they had made an error.

### Data extraction and quality assessment

Data extraction was independently performed by two investigators (SY and ZL); discrepancies were resolved using discussion and consensus. A predefined standardized data extraction form was used to collect data. The following data were collected from each study: the study name, the first author’s name, publication year, the study design, the study location, the patients’ ages, the patients’ sex distribution, the definition of night or weekend, disease severity, adjustments, outcomes, odds ratios (ORs) and 95% CIs, numbers of patients discharged during the nighttime and the daytime, crude hospital mortality among patients discharged during nighttime and daytime, numbers of patients discharged during the weekend and weekdays, crude hospital mortality among patients discharged during the weekend and weekdays, and total number of patients discharged. We also checked the supplementary files and contacted the study authors in cases where more detailed information was needed.

Because all of the included studies were cohort studies, the Newcastle-Ottawa Scale (NOS) was used to assess study quality (available at: http://www.ohri.ca/programs/clinical_epidemiology/oxford.asp) [22]. This scale uses a star system to evaluate study quality in three domains: cohort selection (maximum of four stars), comparability (maximum of two stars), and outcome (maximum of three stars). A score of nine stars indicates the highest possible quality. For the present study, we defined high-quality studies as having >5 stars (a low risk of bias) and low-quality studies as having ≤5 stars (a high risk of bias) [23, 24]. Two investigators (SY and ZL) independently performed the quality assessment; discrepancies were resolved using discussion and consensus.

### Statistical analysis

The primary meta-analysis evaluated the association between nighttime ICU discharge and hospital mortality. The secondary meta-analysis evaluated the association between weekend ICU discharge and hospital mortality. The overall estimates were presented as OR and 95% CI values, which were determined using a random-effects model that accounted for any differences between the studies, even if there was no statistically significant heterogeneity [25].

The individual estimates were used in the sub-analyses for one study [12] that separately reported ORs for weekday night discharge, weekend daytime discharge, weekend night discharge, and weekday daytime discharge. However, to compare nighttime and daytime discharge, we combined the weekend night discharges and weekday night discharges into a single group. An overall estimate for this group was calculated from the available ORs for weekend night discharge and weekday night discharge using a fixed-effects model and the inverse-variance method. To compare weekend and weekday discharge, we combined weekend night discharges and weekend day discharges into another group. An overall estimate for this group was calculated from the available ORs for weekend night discharge and weekend day discharge using a fixed-effects model and the inverse-variance method [26].

Heterogeneity was evaluated using the Cochran *Q* statistic and *I* ^2^ statistic, which are quantitative measures of inconsistency across studies [27], and heterogeneity was considered statistically significant at *P* values <0.1 or *I* ^2^ values >50%. Subgroup analyses were performed to examine the potential sources of heterogeneity using pre-specified subgroups that included geographical region, study design, and study characteristics (definition of nighttime and the total number of patients discharged).

We also performed post-hoc subgroup analyses according to adjustments for certain confounding factors (adjustment for illness severity at the ICU discharge, adjustment for treatment limitation orders, and adjustment for premature discharge), as these confounding factors might affect the results of our analyses.

Sensitivity analysis was performed to explore the possible causes of any heterogeneity and to estimate the influence of missing studies on the overall estimates by changing the pooling model (from a random-effects model to a fixed-effects model) and using the one-study-out method. Egger linear regression testing was performed to test for publication bias [28]. All statistical analyses were performed using STATA software (version 12.1; StataCorp, College Station, TX, USA), and differences were considered statistically significant at a two-tailed *P* value <0.05.

## Results

### Study selection and study characteristics

**Fig. 1:**
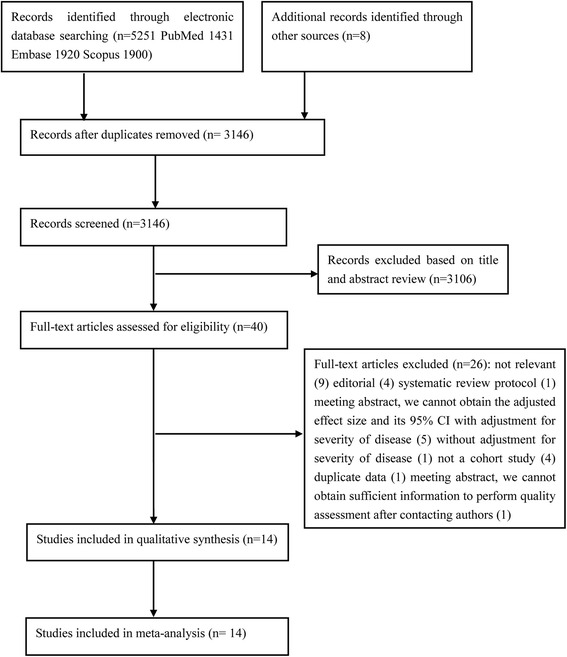
Flow chart of the article selection process

The initial search identified 5259 potentially relevant publications, although 2113 reports were excluded because of duplicate publication. We also excluded 3106 studies based on reviews of the titles and abstracts. Full-text reviews were performed for the remaining 40 studies, and we ultimately identified 14 cohort studies for inclusion in the meta-analysis [5–15, 29–31]. The justifications for the study exclusions are shown in Additional file 1: Table S2. The strategies for study identification and study selection are shown in Fig. 1.

The main characteristics of the 14 included studies are shown in Tables 1, 2 and 3. The 14 studies were all published in English between 2000 and 2015. Six studies were performed in Oceania [7–10, 15, 30], four studies were performed in Europe [5, 6, 14, 29], and the other four studies were performed in North America [11–13, 31]. The studies included six single-center studies and eight multicenter studies, and used a retrospective design (n = 12) or a prospective design (n = 2).

**Table 1:** The main characteristics of the cohort studies included in this meta-analysis

First author/publication year	Study location	Study design	Age (years)	Male (%)	Definition of night or weekend	Disease severity	Population	Adjustments	Outcome	OR and 95% CIs	NOS score	Reference
Santamaria et al./2015	40 ICUs in Australia and New Zealand	Prospective multicenter cohort	Median (IQR) 63 (49–74)	61	Night (18:00–06:00)	APACHE III-J risk of death median (IQR) 0.09 (0.03–0.25)	Adult	Markers of illness severity at the time of ICU discharge: age, cardiac surgery, treatment limitation order, tracheostomy, ongoing dialysis, parenteral nutrition, and altered conscious state	In-hospital mortality	1.16 (0.89, 1.53)	8	[15]
Azevedo et al./2015	5 ICUs in Canada	Retrospective multicenter cohort	57.5 (18.0)	57.9	Night (19:00–07:59), weekend (Fri 19:00–Mon 07:59)	APACHE II score 19.4 (7.5)	Adult	Demographics, co-morbidity, APACHE II score at ICU admission, use of mechanical ventilation, ICU length-of-stay, surgical status, admission source, primary diagnostic category, study year, type of hospital	In-hospital mortality	Night 1.29 (1.14, 1.46), weekend 0.95 (0.84, 1.07)	6	[31]
Gantner et al./2014	103 ICUs contributing to ANZICS APD from 2005 to 2012	Retrospective multicenter cohort	After-hours 59.4 (19.8), in-hours 60.3 (19.3)	NA	Night (18:00–06:00)	APACHE III score After-hours 50.0 (25.3), in-hours 46.5 (22.9)	Adult	APACHE III risk of death at ICU admission, presence of treatment limitation orders at ICU admission, diagnostic category, hospital site	In-hospital mortality	1.34 (1.30, 1.38)	7	[30]
Laupland et al./2011	French ICUs The Outcomerea database	Retrospective multicenter cohort	Median (IQR) 62 (49–75)	61	Night 18:00-07:59)	SAPS II score median (IQR) 40 (28–56)	Adult	Admission SAPSII, medical/surgical classification, presence of septic shock, admission decision to forego life-sustaining therapy(DFLST) order, discharge SOFA score	In-hospital mortality	1.54 (1.12, 2.11)	7	[29]
Singh et al./2010	1 ICU in a tertiary care teaching hospital in Australia	Retrospective single-center cohort	Median 60	61.3	Night (18:00–07:59), weekend (Sat and Sun)	APACHE II score median 18 (range, 1–44)	Adult	Age, APACHE II score at ICU admission, discharge destination	In-hospital mortality	Night 1.38 (1.01, 1.88), weekend 1.04 (0.73, 1.46)	6	[10]
Hanane et al./2008	3 ICUs of Mayo Medical Center in USA	Retrospective single-center cohort	Night 61.6 (18.0), day 62.7 (17.8)	Night 56.0, day 53.1	Night (19:00–06:59)	APACHE III score night 47.6 (21.1), day 44.9 (19.0)	Adult	DNR order by the time of transfer, the last ICU day APACHE III predicted mortality	In-hospital mortality	1.05 (0.64, 1.70)	7	[13]
Laupland et al./2008	4 ICUs in the Calgary Health Region, Alberta, Canada	Retrospective single-center cohort	Median (IQR) 63.7 (49.9–73.8)	64	Night (18:00–07:59), weekend (Sat and Sun)	APACHE II score 25.1 (8.48)	Adult	Noncardiac surgery, cardiac surgery, age, APACHE II at ICU admission, weekend admission, night admission, regional resident	In-hospital mortality	Weekday night discharge 1.20 (1.01, 1.41), weekend day discharge 0.81 (0.67, 0.98), weekend night discharge 1.35 (1.05, 1.73)	6	[12]
Pilcher et al./2007	40 ICUs in Australia and New Zealand	Retrospective multicenter cohort	Night 58.6 (0.08), day: 59.1 (0.17)	NA	Night (18:00–06:00)	APACHE III score night 47.7 (0.2), day: 46.0 (0.1)	Adult	APACHE III risk of death at admission, emergency admission to ICU	In-hospital mortality	1.42 (1.32, 1.53)	6	[9]
Tobin et al./2006	1 ICU in Australia	Retrospective single-center cohort	64 (13–98)	65	Night (22:00–06:59), weekend (Fri 18:00–Mon 07:59)	APACHE II score median 13 (range 0–53)	Adult	Age, APACHE II score at admission, origin of admission, treatment category	In-hospital mortality	Night 1.63 (1.03, 2.57), weekend 1.46 (1.18, 1.81)	6	[8]
Priestap et al./2006	31 Critical care units across Canada	Retrospective multicenter cohort	Night 61.6 (17.7), day 61.7 (17.5)	Night 58, day 57.4	Night (21:00–06:59)	APACHE II score night 15.7 (7.7), day 15.0 (7.4)	Adult	Differences in illness severity at admission, gender, age, admission source, admission diagnosis, site	In-hospital mortality	1.22 (1.10, 1.36)	6	[11]
Duke et al./2004	1 ICU in the Northern Hospital in Australia	Prospective single-center cohort	Median (IQR) 62 (42–73)	NA	Night (22:00–07:30)	APACHE II score 15 (10–21)	Adult	age, APACHE II predicted mortality at admission, premature discharge, delayed discharge, limitation of medical treatment decision, emergency admission, mechanical ventilation, APACHE II diagnosis, chronic health status categories	In-hospital mortality	1.7 (1.03, 2.9)	7	[7]
Uusaro et al./2003	18 ICUs in university and central hospitals in Finland	Retrospective multicenter cohort	NA	NA	Night (16:00–08:00), weekend (Fri 16:00–Sun 24:00)	SAPS II score 34 (17)	Adult	Disease severity at ICU admission, intensity of care, and whether restrictions for future care were set	In-hospital mortality	Night 1.11 (0.93, 1.31), weekend 0.88 (0.73, 1.07)	7	[14]
Beck et al./2002	9 ICUs in a district general hospital in United Kingdom	Retrospective single-center cohort	57 (19)	61.7	Night (20:00–07:59)	APACHE II probabilities 18.3 (18.7)	Adult	Disease severity at ICU admission	In-hospital mortality	1.70 (1.28, 2.25)	6	[6]
Goldfrad et al./2000	88 ICUs in the United Kingdom	Retrospective multicenter cohort	Mean (95% CI) night 57.5 (56.4–58.7), day 58.2 (57.9–58.5)	NA	Night (22:00–06:59)	APACHE II score mean (95% CI), night 15.5 (15.1–16.0), day 14.6 (14.5–14.7)	Adult	Case-mix (age, medical history, acute severity), premature discharge	In-hospital mortality	1.17 (0.92, 1.49)	6	[5]

*Abbreviations*: *OR* odds ratio, *CI* confidence interval, *ICU* intensive care unit, *IQR* interquartile range, *APACHE* Acute Physiology and Chronic Health Evaluation, *SAPS* Simplified Acute Physiology Score, *SOFA* sequential organ failure assessment, *DNR* do-not-resuscitate assessment, *ANZICS APD* Australian and New Zealand Intensive Care Society Adult Patient Database, *NOS* Newcastle-Ottawa Scale, *NA* information not available Continuous data given as mean (sd or 95% CI) or median (interquartile range) if provided by the study authors

**Table 2:** Number of patients and crude hospital mortality in studies in which outcomes were assessed for nighttime versus daytime discharge

First author/publication year	Total number of patients discharged	Discharged during daytime, *n* (%)	Discharged during nighttime, *n* (%)	Crude hospital mortality among patients discharged during daytime, %	Crude hospital mortality among patients discharged during nighttime, %
Santamaria et al./2015 [15]	10,211	8539 (83.6)	1672 (16.4)	4.8	7.4
Azevedo et al./2015 [31]	19,622	16,117 (82.1)	3505 (17.9)	8.8	11.8
Gantner et al./2014 [30]	710,535	601,151 (84.6)	109,384 (15.4)	3.6	6.4
Laupland et al./2011 [29]	5992	5333 (89.0)	659 (11.0)	5	9
Singh et al./2010 [10]	1871	1221 (65.3)	650 (34.7)	10.1	13.7
Hanane et al./2008 [13]	11,659	11,241 (96.4)	418 (3.6)	4.5	5.3
Laupland et al./2008 [12]	17,864	14,151 (79.2)	3713 (20.8)	5	12
Pilcher et al./2007 [9]	76,690	62,704 (81.8)	13,986 (18.2)	5.3	8
Tobin et al./2006 [8]	10,903	NA	NA	NA	NA
Priestap et al./2006 [11]	47,062	42,290 (89.9)	4772 (10.1)	9	11.8
Duke et al./2004 [7]	1870	1578 (84.0)	292 (16.0)	4.3	8.2
Uusaro et al./2003 [14]	20,623	16,952 (82.2)	3671 (17.8)	9.8	11.5
Beck et al./2002 [6]	1654	1351 (81.7)	303 (18.3)	11.2	18.8
Goldfrad et al./2000 [5]	16,756	15,747 (94.0)	1009 (6.0)	13	18.1

*NA* information not available

**Table 3:** Number of patients and crude hospital mortality in studies in which outcomes were assessed for weekend versus weekday discharge

First author/publication year	Total number of patients discharged	Discharged during weekend, *n* (%)	Discharged during weekdays, *n* (%)	Crude hospital mortality among patients discharged during weekend, %	Crude hospital mortality among patients discharged during weekdays, %
Azevedo et al./2015 [31]	19,622	4676 (23.8)	14,946 (76.2)	NA	NA
Singh et al./2010 [10]	1871	567 (30.3)	1304 (69.7)	NA	NA
Laupland et al./2008 [12]	17,864	4661 (26.1)	13,203 (73.9)	6	7
Tobin et al./2006 [8]	10,903	NA	NA	NA	NA
Uusaro et al./2003 [14]	20,623	2932 (14.2)	17,691 (85.8)	9.2	10.2

*NA* information not available

Disease severity was reported based on the Acute Physiology and Chronic Health Evaluation (APACHE) II score (n = 8 studies), the APACHE III score (n =4), or the Simplified Acute Physiology Score (SAPS) II (n = 2). In all 14 studies there was adjustment for a wide range of potential confounders, such as age, treatment limitation orders, premature discharge, diagnostic category, and the use of mechanical ventilation. In 11 studies there was adjustment for disease severity on ICU admission [5–12, 14, 30, 31], and in 3 studies adjustment for disease severity on ICU discharge [13, 15, 29]; in 6 studies there was adjustment for treatment limitation orders [7, 13–15, 29, 30], but no such adjustment in the other 8 studies [5, 6, 8–12, 31]. In two studies there was adjustment for premature discharge [5, 7].

A total of 953,312 patients were included in the meta-analysis, with the study samples ranging from 1654 patients to 710,535 patients. Four studies included ≤10,000 patients [6, 7, 10, 29] and 10 studies included >10,000 patients [5, 8, 9, 11–15, 30, 31]. The mean proportion of nighttime discharges in 13 studies was 15.3% (range 3.6–34.7%), and one study did not report this information [8]. The evaluated discharge times included nighttime in all 14 studies [5–15, 29–31] and weekends plus nighttime in 5 studies [8, 10, 12, 14, 31]. None of the 14 studies used consistent definitions of nighttime or weekend. The average NOS score of the included studies was 6.5 (range 6–8) (see Additional file 1: Table S3).

### Nighttime discharge and hospital mortality

**Fig. 2:**
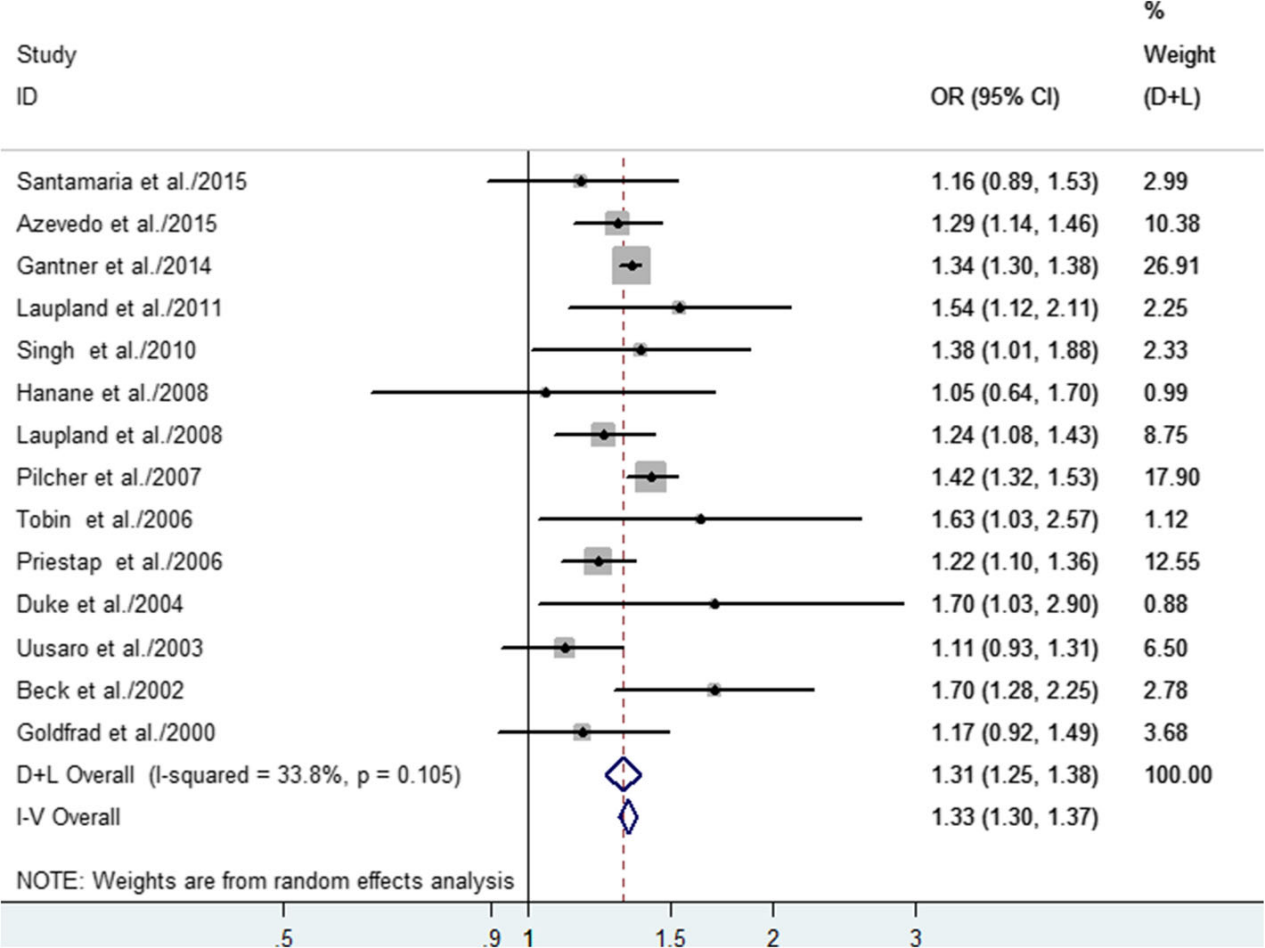
Forest plots of the association between nighttime discharge from the ICU and hospital mortality. The size of each *square* is proportional to the study weight. *Open diamond* represents the overall pooled OR. *D + L* random effects, *I-V* fixed effects

The 14 studies had 953,312 patients who were evaluated for daytime/nighttime discharge (Table 2). The adjusted OR for hospital mortality was significantly higher among patients discharged during the nighttime, compared to patients discharged during the daytime (OR 1.31, 95% CI 1.25–1.38, *P* < 0.0001) (Fig. 2), and the individual studies had low heterogeneity (*I* ^2^ = 33.8%, *P* = 0.105). We also performed subgroup analyses and sensitivity analyses (Table 4), which revealed that the significant association between nighttime discharge and hospital mortality was not substantially modified by geographical region, the total number of discharges, or study design (Additional file 3: Figures S1–S3).

**Table 4:** Subgroup and sensitivity analyses for hospital mortality

Analysis	Studies, *n*	Odds ratio (95% CI)	*P* heterogeneity	*I* ^2^	Study reference
Subgroup analysis					
The definition of night					
18:00–06:00	3	1.36 (1.29, 1.43)	0.198	38.30%	[9, 15, 30]
18:00–07:59	3	1.30 (1.15, 1.46)	0.432	0.00%	[10, 12, 29]
19:00–07:59	1	1.29 (1.14, 1.46)			[31]
19:00–06:59	1	1.05 (0.64, 1.70)			[13]
22:00–07:30	1	1.7(1.03, 2.9)			[7]
21:00–06:59	1	1.22 (1.10, 1.36)			[11]
16:00–08:00	1	1.11(0.93, 1.31)			[14]
20:00–07:59	1	1.70 (1.28, 2.25)			[6]
22:00–06:59	2	1.30 (0.96, 1.76)	0.209	36.80%	[5, 8]
Geographic region					
Oceania	6	1.35 (1.31, 1.39)	0.457	0.00%	[7–10, 15, 30]
Europe	4	1.33 (1.08, 1.63)	0.039	64.00%	[5, 6, 14, 29]
North America	4	1.24 (1.16, 1.33)	0.82	0.00%	[11–13, 31]
Total discharge number					
≤ 10000	4	1.56 (1.32, 1.84)	0.785	0.00%	[6, 7, 10, 29]
> 10000	10	1.29 (1.23, 1.36)	0.091	39.90%	[5, 8, 9, 11–15, 30, 31]
Study design					
Multicenter studies	8	1.30 (1.23, 1.38)	0.067	47.00%	[5, 9, 11, 14, 15, 29–31]
Single-center studies	6	1.38 (1.20, 1.59)	0.268	22.10%	[6–8, 10, 12, 13]
Whether or not adjusted for severity of illness at the time of ICU discharge					
YES	3	1.26 (1.02, 1.57)	0.296	17.80%	[13, 15, 29]
NO	11	1.31 (1.25, 1.38)	0.076	40.90%	[5–12, 14, 30, 31]
Whether or not adjusted for treatment limitation orders					
Yes	6	1.28 (1.15, 1.43)	0.152	38.10%	[7, 13–15, 29, 30]
No	8	1.32 (1.23, 1.42)	0.116	39.40%	[5, 6, 8–12, 31]
whether or not adjusted for premature discharge					
Yes	2	1.31 (0.94, 1.84)	0.2	39.20%	[5, 7]
No	12	1.31 (1.25, 1.38)	0.09	37.70%	[6, 8–15, 29–31]
Sensitivity analysis					
Fixed-effects model	14	1.33 (1.30, 1.37)	0.105	33.80%	[5–15, 29–31]
Random-effects model	14	1.31 (1.25, 1.38)	0.105	33.80%	[5–15, 29–31]
One-study-out method					
Santamaria et al./2015	1	1.31 (1.24, 1.39)			[15]
Azevedo et al./2015	1	1.31 (1.25, 1.37)			[31]
Gantner et al./2014	1	1.31 (1.24, 1.38)			[30]
Laupland et al./2011	1	1.30 (1.21, 1.39)			[29]
Singh et al./2010	1	1.32 (1.25, 1.39)			[10]
Hanane et al./2008	1	1.31 (1.25, 1.38)			[13]
Laupland et al./2008	1	1.32 (1.25, 1.39)			[12]
Pilcher et al./2007	1	1.31 (1.24, 1.37)			[9]
Tobin et al./2006	1	1.29 (1.22, 1.36)			[8]
Priestap et al./2006	1	1.33 (1.26, 1.40)			[11]
Duke et al./2004	1	1.32 (1.25, 1.38)			[7]
Uusaro et al./2003	1	1.31 (1.24, 1.38)			[14]
Beck et al./2002	1	1.31 (1.24, 1.38)			[6]
Goldfrad et al./2000	1	1.33 (1.27, 1.39)			[5]

**Fig. 3:**
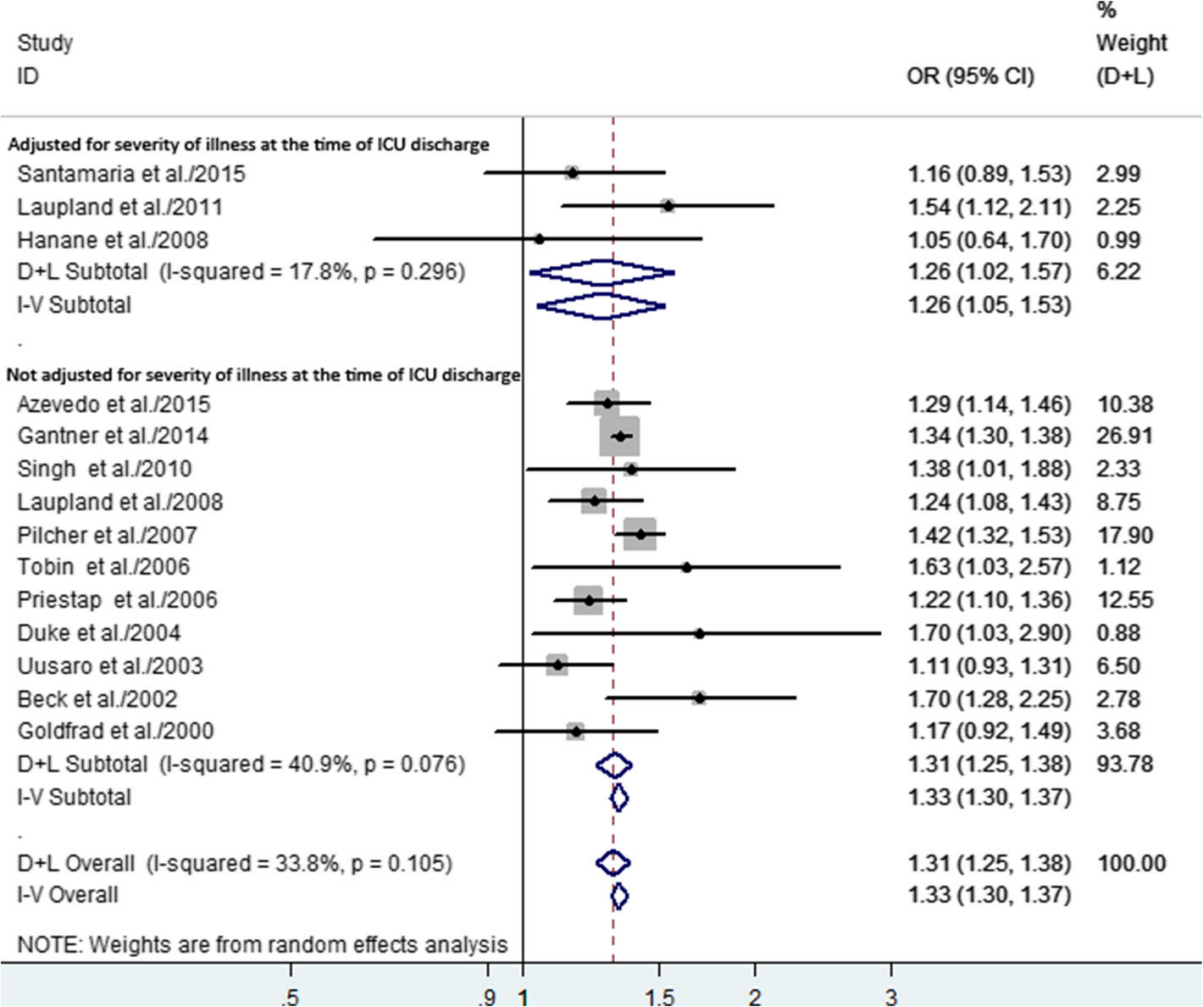
Forest plots of the association between nighttime discharge from the ICU and hospital mortality stratified by whether or not the data were adjusted for severity of illness at the time of ICU discharge. The size of each *square* is proportional to the study weight. *Open diamonds* represent the pooled OR. *D + L* random effects, *I-V* fixed effects

Among the 11 studies in which there was adjustment for disease severity on ICU admission, the summary OR for hospital mortality was 1.31 (95% CI 1.25–1.38). A summary OR was provided for hospital mortality with a broader range (OR 1.26, 95% CI 1.02–1.57) in the three studies in which there was adjustment for disease severity at ICU discharge (Fig. 3). In our meta-analysis, the risk of hospital mortality did not depend on whether or not the study analyses were adjusted for disease severity at ICU discharge.

**Fig. 4:**
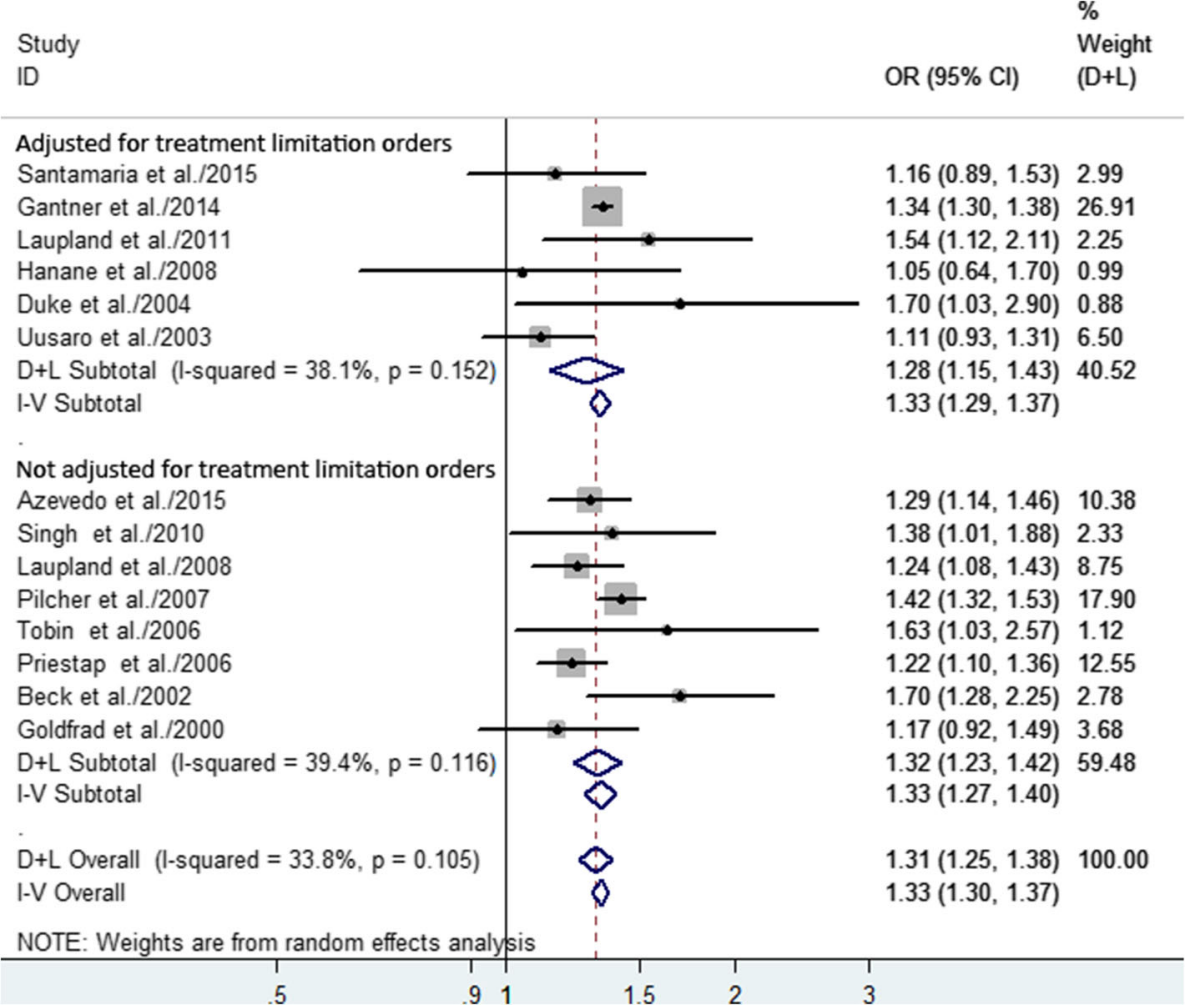
Forest plots of the association between nighttime discharge from the ICU and hospital mortality stratified by whether or not the data were adjusted for treatment limitation orders. The size of each *square* is proportional to the study weight. *Open diamonds* represent the pooled OR. *D + L* random effects, *I-V* fixed effects

**Fig. 5:**
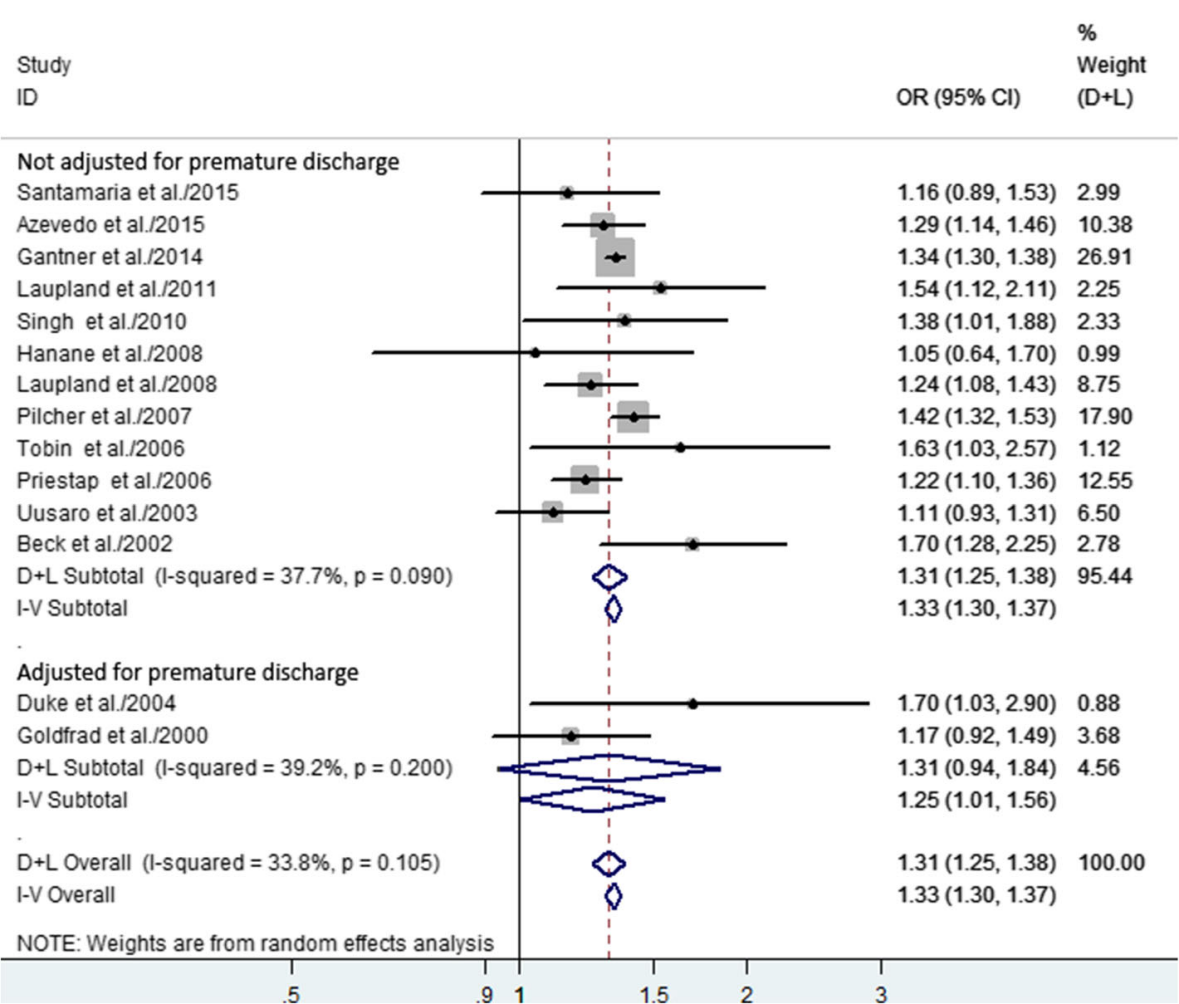
Forest plots of the association between nighttime discharge from the ICU and hospital mortality stratified by whether or not the data were adjusted for premature discharge. The size of each *square* is proportional to the study weight. *Open diamonds* represent the pooled OR. *D + L* random effects, *I-V* fixed effects

Among the six studies in which there was adjustment for treatment limitation orders, the summary OR for hospital mortality was 1.28 (95% CI 1.15–1.43); among the remaining eight studies without this adjustment, the summary OR for hospital mortality was 1.32 (95% CI 1.23–1.42) (Fig. 4). In our meta-analysis, the risk of hospital mortality did not depend on whether or not there was adjustment for treatment limitation orders. We did not observe a significant relationship between nighttime discharge and hospital mortality in the subgroup analysis that was adjusted for premature discharge (Fig. 5). Our sensitivity analyses suggested that the overall estimates were not materially altered by changing the pooling models (random-effects model, OR 1.31, 95% CI 1.25–1.38; fixed-effects model, OR 1.33, 95% CI 1.30–1.37) and were not materially altered when an individual study was omitted from the sequence, with a range of 1.29 (95% CI 1.22–1.36) to 1.33 (95% CI 1.27–1.39) (Table 4).

### Weekend discharge and hospital mortality

**Fig. 6:**
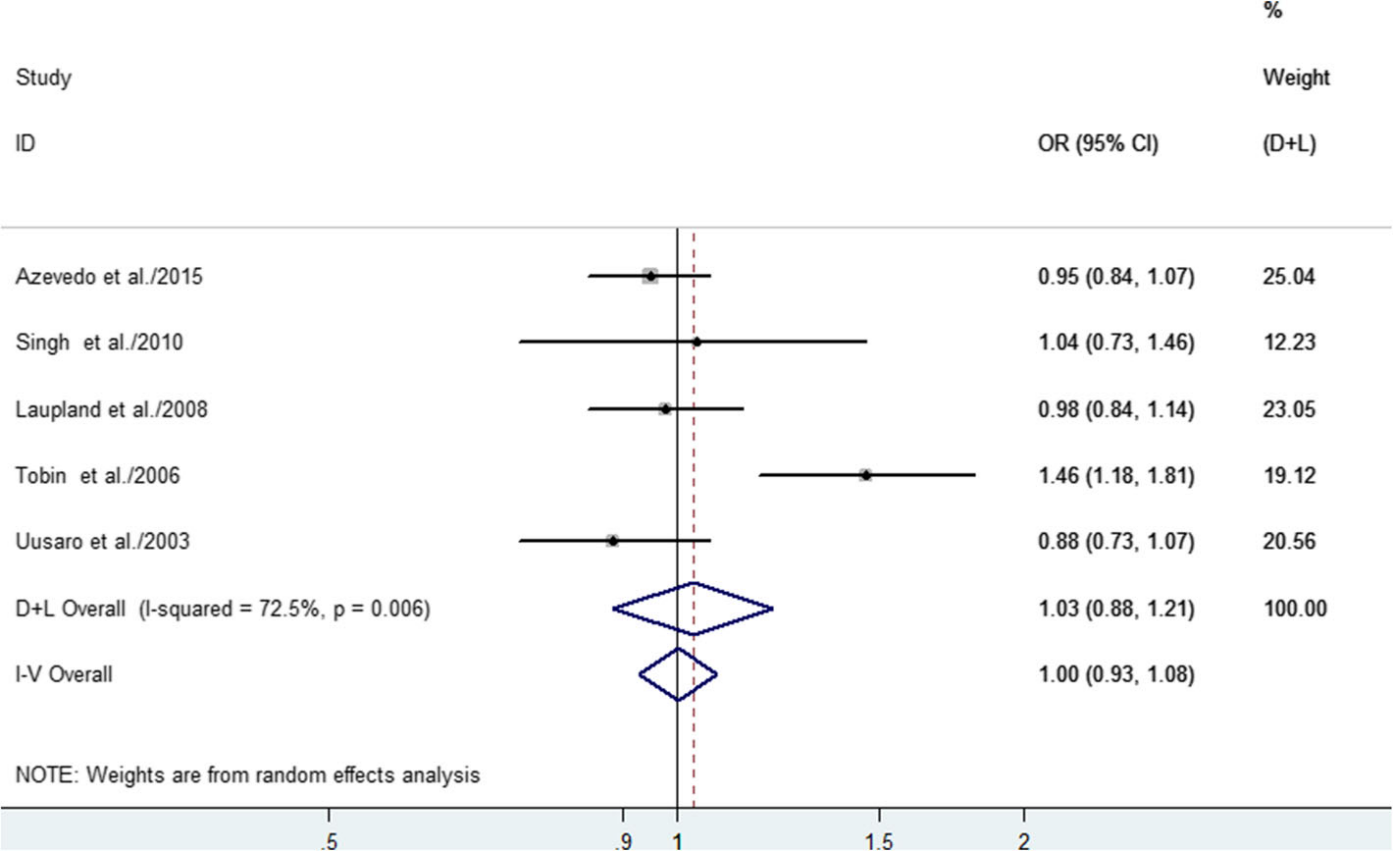
Forest plots of the association between weekend discharge from the ICU and hospital mortality. The size of each square is proportional to the study weight. *Open diamond* represents the overall pooled OR. *D + L* random effects, *I-V* fixed effects

Five studies with 70,883 patients evaluated weekday/weekend discharges (Table 3). There was no difference in the adjusted ORs for hospital mortality when we compared patients who were discharged during the weekend or on weekdays (OR 1.03, 95% CI 0.88–1.21, *P* = 0.68) (Fig. 6). However, the studies exhibited significant heterogeneity (*I* ^2^ = 72.5%, *P* = 0.006), and we performed sensitivity analyses to explore the possible explanations for the heterogeneity. Our sensitivity analyses suggested that the overall estimates were not materially altered by changing the pooling models (random-effects model, OR 1.03, 95% CI 0.88–1.21; fixed-effects model, OR 1.00, 95% CI 0.93–1.08) and were not materially altered when an individual study was omitted from the sequence, with a range of 0.95 (95% CI 0.87–1.03) to 1.08 (95% CI 0.89–1.31). We were unable to identify the specific study that caused the heterogeneity, which might have been explained by the different patient populations, weekend definitions (including a different number of weekend days and weekend nights), and adjustments for confounding factors. However, when the Tobin study [8] was omitted, the heterogeneity among the remaining four studies was markedly reduced (*I* ^2^ = 0.0%, *P* = 0.792), and we did not observe an association between weekend discharge and hospital mortality.

### Publication bias

Egger’s test did not reveal any significant evidence of publication bias (*P* = 0.662 for nighttime studies, *P* = 0.507 for weekend studies) (Additional file 3: Figures S4 and S5).

## Discussion

The present study revealed that nighttime ICU discharge is associated with an increased risk of hospital mortality compared to daytime ICU discharge. Furthermore, it seems that nighttime ICU discharge was also associated with an increased risk of hospital mortality in the subgroup analyses that were stratified according to geographical region, the total number of discharges, or study design (multicenter or single-center).

Patient-related factors, such as disease severity and limitation of medical treatment, are considered crucial determinants of mortality after nighttime ICU discharge [7, 15], and disease severity at the ICU admission or discharge is useful for predicting post-ICU mortality. Some of the studies in our meta-analysis found that patients discharged at nighttime were typically older and had more severe injuries, co-morbidities, or multi-organ dysfunction, compared to patients with a daytime discharge, which may explain the higher mortality with nighttime ICU discharge [10, 29, 30]. However, our methodology was initially based on precluding the confounding effect of disease severity. Three studies included adjustment for disease severity at ICU discharge, and the remaining 11 studies included adjustment for disease severity at ICU admission, and both sets had acceptable heterogeneity. After adjusting for disease severity at ICU discharge or admission, there was still a significant relationship between nighttime discharge and hospital mortality (Fig. 3).

Limitation of medical treatment orders refers to limiting or withholding one or more life-support therapies, such as cardiopulmonary resuscitation (do-not-resuscitate orders) or the use of palliative care measures [7]. Patients discharged at nighttime were more likely to have a medical treatment limitation order, which was found to be an independent predictor of mortality in previous studies [3, 7, 13, 15]. For example, Santamaria et al. found that treatment limitation orders were an independent predictor of hospital survival (OR 35.4, 95% CI 27.5–45.6). However, Ouanes et al. observed a relationship between nighttime discharge and 7-day mortality or readmission after excluding patients with treatment limitation orders [32]. In our meta-analysis, adjustment for treatment limitation orders was documented in six studies, and nighttime discharge remained associated with an increased risk of hospital mortality after the adjustment.

Poor-quality medical care (i.e., lower staffing levels, lower nurse-to-patient ratios, and less surveillance) may partly explain the association between nighttime discharge and increased mortality. Staffing levels and nurse-to-patient ratios in the ICU and general wards are invariably lower at night, and previous studies have reported that increased mortality is associated with decreased staffing levels in the ICU and general wards [33, 34]. In addition, monitoring devices and life-sustaining devices are considered less immediately available in the general wards [35], and 64% of major adverse events that occur within 72 h after ICU discharge may be predicted and prevented by monitoring the patient’s vital signs (e.g., an abnormal respiratory rate or tachycardia) [36, 37]. Moreover, transfer to a ward is associated with increased post-ICU mortality, while transfer to a high-dependency unit is not, which suggests that patients’ outcomes may be associated with the intensity of post-ICU medical care [6].

There are also systemic factors that may contribute to the post-ICU mortality rate. Practical guidelines recommend that ICU patients should be constantly evaluated for discharge based on their physiological status and the necessity of ICU monitoring [38]. However, in practice, patients are rarely evaluated for discharge at night, unless the ICU is facing pressure from additional admissions. This dilemma may lead to premature discharge, which refers to an unplanned transfer of a patient to make an ICU bed available for a more acutely or seriously sick patient [31].

The proportions of premature discharges vary among different studies based on their defining criteria. Premature ICU discharge was an independent risk factor for mortality in some studies [5, 39], and an additional 48 h of ICU stay may provide a 39% reduction in mortality among high-risk patients [40, 41]. Two of the included studies included adjustment for premature discharge, and we did not observe a significant relationship between nighttime discharge and hospital mortality in this subgroup [5, 7]. However, in these two studies (by Goldfrad et al. [5] and Duke et al. [7]) there are conflicting results, and mild heterogeneity further obscures the results of this subgroup analysis (*I* ^2^ = 39.2%). Another study by Santamaria et al. also did not identify an association between premature discharge and subsequent mortality [15]. Thus, the current studies have inconsistent results on whether premature discharge is an independent risk factor for post-ICU mortality, and whether premature discharge affects the association between nighttime ICU discharge and hospital mortality and the magnitude of any related effects remains unclear.

Delayed discharge refers to a planned or prepared discharge that is delayed for some reason, which is commonly a lack of ward beds [42]. Similar to premature discharge, delayed discharge is more likely to occur at night, and each 1-h delay is estimated to be associated with an adjusted 3% increase in the risk of mortality [43, 44]. Delayed discharge may increase the risk of ICU-acquired infections, which independently influence post-ICU mortality and lead to additional delayed discharges [45].

Both delayed discharge and premature discharge are thought to reflect a limited bed capacity, and some authors even consider nighttime discharge as a marker of a bed shortage [11, 32]. This may partially reflect the increasing trend in the ratio of nighttime-t-daytime discharge during recent years. Although the average bed occupancy rate in American ICUs has remained fairly constant (1985–2000, 65%; 2000–2010, 68%), there has been an increase in the demand for intensive care practitioners to provide critical care services [46–48]. Interestingly, improving the number of beds does not always reduce delayed discharge, as Williams et al. found that delayed discharges increased by 4%, despite a significant increase in bed capacity (2000–2001 vs. 2008), which reduced the proportion of “no-bed delays” from 74% to 36% [49]. This finding suggests that the problem does not lie in the number of beds, but rather in the inability of the ward to accept patients who are discharged from the ICU in a timely manner. In this scenario, nighttime discharge may be considered an indicator of inefficient patient delivery [50], with nighttime discharge serving to optimize the use of existing ICU beds, instead of simply enlarging the ICU [15, 50].

We did not find an association between weekend discharge and hospital mortality, although heterogeneity among the studies may be a question. The different patient populations, weekend definitions (including a different number of weekend days and weekend nights), statistical analyses, and adjustments for confounding factors may also explain the significant heterogeneity. For example, the only positive result (from Tobin et al. [8]) was based on a univariate analysis, while the other studies used a multivariate or logistic regression model. In addition, Tobin et al. defined weekends as being from 18:00 on Friday to 07:59 on Monday (3 days and 3 nights), while the other studies defined weekends as only Saturday and Sunday [10, 12]. Moreover, the fact that weekend staffing levels and medical care resources are similar to weekdays might explain the negative association between weekend discharge and hospital mortality, although this conclusion remains speculative [12].

### Strengths and limitations

This study has several strengths. First, we identified studies using a comprehensive systematic literature search. Second, we evaluated 953,312 patients with daytime/nighttime discharge, and the large sample size significantly increased the statistical power of the analysis. Third, the pooled estimates were stable after the comprehensive sensitivity analyses. Fourth, we did not detect publication bias, which indicates that the pooled estimates may be unbiased.

This study also has several limitations. First, the cohort studies were observational and descriptive, and we cannot comment on the causality of the relationships between hospital mortality and the factors that we evaluated. Second, the studies used different definitions for nighttime and weekend, which inevitably introduces heterogeneity. Third, although both disease severity at discharge and admission can predict post-ICU mortality, only three of the included studies measured disease severity at ICU discharge. Premature ICU discharge was also more common at night, although only two of the included studies adjusted for premature discharge. Thus, the limited numbers of studies that adjusted for premature discharge or disease severity at ICU discharge might cause residual confounding. Fourth, none of the studies reported the exact demand of care and actual level of care in the ward, which obscures whether or how treatment and nursing care insufficiencies might influence post-ICU mortality. Last, the included studies did not report long-term prognosis or its possible relationship with nighttime discharge, which may also be an important issue. Moreover, the studies failed to address novel trends in critical care, including tele-ICU, liaison nurses, and rapid response teams, which may profoundly alter intensive care and post-ICU care and further alter the rate and effect of nighttime discharge. Therefore, the generalizability of our results in the real world must be tested in future studies [51].

## Conclusions

The present meta-analysis revealed that nighttime ICU discharge was associated with an increased risk of hospital mortality, compared to daytime discharge. Although we did not detect a significant association between hospital mortality and weekend ICU discharge, there was significant heterogeneity among the included studies. Thus, these conclusions should be interpreted with caution, and further large-scale, well-designed, multicenter prospective studies are needed to improve our understanding of the association between ICU discharge times and hospital mortality.

## Additional files

Additional file 1: Table S1. Database search strategies. **Table S2.** The justifications for the study exclusions (n = 26). **Table S3.** Newcastle-Ottawa quality assessment of included studies. **Table S4**. The main characteristics of the excluded meeting abstracts. (DOCX 98 kb)
Additional file 2: PRISMA 2009 checklist. (DOC 62 kb)
Additional file 3: Figure S1. Forest plots of the association between nighttime discharge from the ICU and hospital mortality stratified by geographic region. The size of each *square* is proportional to the study weight. *Open diamonds* represent the pooled OR. *D + L* refers to random effects and *I-V* to fixed effects. **Figure S2.** Forest plots of the association between nighttime discharge from the ICU and hospital mortality stratified by study design. The size of each *square* is proportional to the study weight. *Open diamonds* represent the pooled OR. *D + L* refers to random effects and *I-V* to fixed effects. **Figure S3.** Forest plots of the association between nighttime discharge from the ICU and hospital mortality stratified by the total discharge number. The size of each *square* is proportional to the study weight. *Open diamonds* represent the pooled OR. *D + L* refers to random effects and *I-V* to fixed effects. **Figure S4.** Funnel plots showing the association of nighttime discharge from the ICU with hospital mortality. *s.e.* refers to standard error, or refers to odds ratio. **Figure S5.** Funnel plots showing the association of weekend discharge from the ICU with hospital mortality. *s.e.* refers to standard error, or refers to odds ratio. (ZIP 395 kb)

## Abbreviations

APACHE: Acute Physiology and Chronic Health Evaluation
CI: confidence interval
DNR: do-not-resuscitate assessment
ICU: intensive care unit
IQR: interquartile range
NA: information not available
OR: odds ratio
SAPS: Simplified Acute Physiology Score
SOFA: sequential organ failure assessment
