# Supplementary material for: Association between time of discharge from ICU and hospital mortality: a systematic review and meta-analysis
Source: Crit Care. 2016 Dec 1;20:390. doi: 10.1186/s13054-016-1569-x (PMC5131545; doi:10.1186/s13054-016-1569-x)
Supplement: Additional file 1: Table S1. — Database search strategies. Table S2. The justifications for the study exclusions (n = 26). Table S3. Newcastle-Ottawa quality assessment of included studies. Table S4. The main characteristics of the excluded meeting abstracts. (DOCX 98 kb) [file 13054_2016_1569_MOESM1_ESM.docx]

**Table S1.** Database search strategies (1-Aug-2016).

**PubMed database**

***Search strategy***

1. intensive care unit
2. intensive care units
3. ICU
4. ICUs
5. Intensive Care Units[MeSH Terms]
6. or/1-5
7. night-shift
8. night
9. nighttime
10. out-of-hours
11. evening
12. off-hour
13. after-hours
14. time
15. time[MeSH Terms]
16. or/7-15
17. discharge
18. discharges
19. transfer
20. transfers
21. Patient Discharge[MeSH Terms]
22. or/17-21
23. mortality
24. Mortality[MeSH Terms]
25. death
26. death[MeSH Terms]
27. or/23-26
28. 6 and 16 and 22 and 27
29. child
30. children
31. infant
32. Child[MeSH Terms]
33. Infant[MeSH Terms]
34. or/29-33
35. 28 not 34
36. animals
37. 35 not 36

**Research results**

| **Searches** | **Results** | **Search Type** | **Actions** |
| --- | --- | --- | --- |
| 1 | intensive care unit [Title/Abstract] | 69430 | Advanced |
| 2 | intensive care units [Title/Abstract] | 17691 | Advanced |
| 3 | ICU [Title/Abstract] | 37825 | Advanced |
| 4 | ICUs [Title/Abstract] | 6878 | Advanced |
| 5 | Intensive Care Units[MeSH Terms] | 63674 | Advanced |
| 6 | or/1-5 | 125265 | Advanced |
| 7 | night-shift [Title/Abstract] | 1461 | Advanced |
| 8 | night [Title/Abstract] | 51745 | Advanced |
| 9 | nighttime [Title/Abstract] | 7383 | Advanced |
| 10 | out-of-hours [Title/Abstract] | 1350 | Advanced |
| 11 | evening [Title/Abstract] | 15089 | Advanced |
| 12 | off-hour [Title/Abstract] | 64 | Advanced |
| 13 | after-hours [Title/Abstract] | 1012 | Advanced |
| 14 | time [Title/Abstract] | 2379756 | Advanced |
| 15 | time[MeSH Terms] | 1205921 | Advanced |
| 16 | or/7-15 | 3326786 | Advanced |
| 17 | discharge [Title/Abstract] | 139024 | Advanced |
| 18 | discharges [Title/Abstract] | 25528 | Advanced |
| 19 | transfer [Title/Abstract] | 339069 | Advanced |
| 20 | transfers [Title/Abstract] | 19629 | Advanced c |
| 21 | Patient Discharge[MeSH Terms] | 22083 | Advanced |
| 22 | or/17-21 | 512474 | Advanced |
| 23 | mortality [Title/Abstract] | 563152 | Advanced |
| 24 | Mortality[MeSH Terms] | 310983 | Advanced |
| 25 | death [Title/Abstract] | 552158 | Advanced |
| 26 | death[MeSH Terms] | 129601 | Advanced |
| 27 | or/23-26 | 1259292 | Advanced |
| 28 | 6 and 16 and 22 and 27 | 1858 | Advanced |
| 29 | child [Title/Abstract] | 319665 | Advanced |
| 30 | children [Title/Abstract] | 839991 | Advanced |
| 31 | infant [Title/Abstract] | 188038 | Advanced |
| 32 | Child[MeSH Terms] | 1662822 | Advanced |
| 33 | Infant[MeSH Terms] | 1006143 | Advanced |
| 34 | or/29-33 | 2425478 | Advanced |
| 35 | 28 not 34 | 1435 | Advanced |
| 36 | animals [Title/Abstract] | 608484 | Advanced |
| 37 | 35 not 36 | 1431 | Advanced |

**Embase database**

***Search strategy***

1. intensive care unit
2. intensive care units
3. ICU
4. ICUs
5. 'intensive care unit'/exp
6. or/1-5
7. night-shift
8. night
9. nighttime
10. out-of-hours
11. evening
12. off-hour
13. after-hours
14. time
15. or/7-14
16. discharge
17. discharges
18. transfer
19. transfers
20. or/16-19
21. mortality
22. 'mortality'/exp
23. death
24. or/21-23
25. 6 and 15 and 20 and 24
26. child
27. children
28. infant
29. 'child'/exp
30. 'newborn'/exp
31. or/26-30
32. 25 not 31
33. 32 AND [humans]/lim

**Search results**

| **No.** | **Query** | **Results** |
| --- | --- | --- |

33 #32 AND [humans]/lim 1920

32 #25 NOT #31 2166

31 #26 OR #27 OR #28 OR #29 OR #30 1742503

30 'newborn'/exp AND [embase]/lim 290967

29 'child'/exp AND [embase]/lim 1461504

28 'infant':ab,ti AND [embase]/lim 122901

27 'children':ab,ti AND [embase]/lim 807906

26 'child':ab,ti AND [embase]/lim 262358

25 #6 AND #15AND #20 AND #24 2548

24 #21 OR #22 OR #23 1323331

23 'death':ab,ti AND [embase]/lim 618713

22 'mortality'/exp AND [embase]/lim 666361

21 'mortality':ab,ti AND [embase]/lim 656747

20 #16 OR #17 OR #18 OR #19 492045

19 'transfers':ab,ti AND [embase]/lim 19211

18 'transfer':ab,ti AND [embase]/lim 293002

17 'discharge':ab,ti AND [embase]/lim 170246

16 'discharges':ab,ti AND [embase]/lim 30658

15 #7 OR #8 OR #9 OR #10 OR #11 OR #12 OR #13 OR #14 2587182

14 'time':ab,ti AND [embase]/lim 2533023

13 'after-hours':ab,ti AND [embase]/lim 970

12 'off-hour':ab,ti AND [embase]/lim 120

11 'evening':ab,ti AND [embase]/lim 17410

10 'out-of-hours':ab,ti AND [embase]/lim 1851

9 'nighttime':ab,ti AND [embase]/lim 8865

8 'night':ab,ti AND [embase]/lim 60487

7 'night shift':ab,ti AND [embase]/lim 1613

6 #1 OR #2 OR #3 OR #4 OR #5 158870

5 'intensive care unit'/exp AND [embase]/lim 94892

4 'icus':ab,ti AND [embase]/lim 9359

3 'icu':ab,ti AND [embase]/lim 65400

2 'intensive care units':ab,ti AND [embase]/lim 19130

1 'intensive care unit':ab,ti AND [embase]/lim 80979

**Scopus database**

***Search strategy***

1. intensive care unit
2. intensive care units
3. ICU
4. ICUs
5. or/1-4
6. night-shift
7. night
8. nighttime
9. out-of-hours
10. evening
11. off-hour
12. after-hours
13. time
14. or/6-13
15. discharge
16. discharges
17. transfer
18. transfers
19. or/15-18
20. mortality
21. death
22. or/20-21
23. 5 and 14 and 19 and 22
24. child
25. children
26. infant
27. or/24-26
28. 23 not 27
29. animals
30. 28 not 29

**Search results**

| **No.** | **Query** | **Results** |
| --- | --- | --- |

30 28 not 29 1900

29 TITLE-ABS-KEY ( "animals " ) 6,610,097

28 23 not 27 1,921

27 TITLE-ABS-KEY ( "infant" ) 1,194,448

25 TITLE-ABS-KEY("children") 2611999

24 TITLE-ABS-KEY("child") 2,611,971

23 #5 AND #14AND #19 AND #22 2518

22 #20 OR #21 1,800,102

21 TITLE-ABS-KEY("death") 993,847

20 TITLE-ABS-KEY("Mortality") 1,054,445

19 #15 OR #16 OR #17 OR #18 2,106,125

18 TITLE-ABS-KEY("transfer") 1,586,180

17 TITLE-ABS-KEY("transfers") 1,586,180

16 TITLE-ABS-KEY("discharges") 538456

15 TITLE-ABS-KEY("discharge") 538456

14 #6 OR #7 OR #8 OR #9 OR #10 OR #11 OR #12 OR #13 9,068,225

13 TITLE-ABS-KEY("time") 8,957,092

12 TITLE-ABS-KEY("After-hours") 2454

11 TITLE-ABS-KEY("off-hour") 406

10 TITLE-ABS-KEY("evening") 34736

9 TITLE-ABS-KEY("out-of-hours") 1718

8 TITLE-ABS-KEY("nighttime") 18234

7 TITLE-ABS-KEY("Night") 134751

6 TITLE-ABS-KEY("Night-shift") 2708

5 #1 OR #2 OR #3 OR #4 162,201

4 TITLE-ABS-KEY("ICUS") 7,912

3 TITLE-ABS-KEY("ICU") 46,930

2 TITLE-ABS-KEY("intensive care units") 151,059

1 TITLE-ABS-KEY("intensive care unit") 151059

**Additional searches**

Two clinical trial registries

http://www.clinicaltrials.gov/

http://www.controlled-trials.com

**Search results**: NO relevant study.

Abstracts from major international conferences were searched by hand in their official journal websites

Society of Critical Care Medicine: Critical Care Medicine [1998–2015]; American Thoracic Society: American Journal of Respiratory and Critical Care Medicine[2009–2016]; European Society of Intensive Care Medicine: Intensive Care Medicine [1988–2014]; International Symposium on Intensive Care and Emergency Medicine: Critical Care [1997–2016]; American College of Chest Physicians: Chest[2003–2015]; Australian and New Zealand Intensive Care Society Annual Meeting: Anaesthesia and Intensive Care [1990–2015]

**Search results**: 8 meeting abstracts were identified*.*

1.YL Bramma, R Allan, R Sundaram. Out-of-hours discharge from the ICU: defining the out-of-hours period and its effect on mortality. Critical Care 2012, 16(Suppl 1):P512 (doi: 10.1186/cc11119)

2.Edie S , Burt K, Paddle J. Association between out-of-hours discharge and mortality in adult patients leaving critical care. Critical Care 2015, 19(Suppl 1):P564 (doi: 10.1186/cc14644)

## 3.Ahmed M. Lumley G, Nourse S. et al. Out-of-hours discharge from critical care: does it matter? Crit Care (2015) 19: P526. doi:10.1186/cc14606

## 4.Gopal S, Terry L, Corbett C. Association between out of hours discharge from the ICU and subsequent readmission. Crit Care (2010) 14: P476. doi:10.1186/cc8708

5.Perry J, Hines H. ‘sooner rather than later’: how delayed discharge from critical care leads to increased out of hours discharges and subsequent increase in in-hospital mortality. Crit Care 2016 20(Suppl 2): P395

6.Harvey, S., Wunsch, H., Welch, C., Harrison D., Rowan K. Hospital mortality associated with day and time of discharge from intensive care units in the United Kingdom. Crit Care (2005) 9: P238. doi:10.1186/cc3301

## 7.Bristlow PJ. Discharge out of hours are associated with increased mortality. Anaesthsia Intensive Care 2003;31:222.

8.Karnik A, Fraser J, Cook D. Out-of-hours discharge: are we risking our patients’ lives? Anaesthesia Intensive Care. 2001;29(2):200

**Hand searching**

*Please note that the lists we present were 14 included studies and 2 reviews; we checked the ref. of these studies (451 records).*

*NO study was identified by hand searching.*

1. Goldfrad C, Rowan K. Consequences of discharges from intensive care at night. Lancet. 2000;355(9210):1138-42.

2. Beck DH, McQuillan P, Smith GB. Waiting for the break of dawn? The effects of discharge time, discharge TISS scores and discharge facility on hospital mortality after intensive care. Intensive care medicine. 2002;28(9):1287-93.

3. Duke GJ, Green JV, Briedis JH. Night-shift discharge from intensive care unit increases the mortality-risk of ICU survivors. Anaesthesia and intensive care. 2004;32(5):697-701.

4. Priestap FA, Martin CM. Impact of intensive care unit discharge time on patient outcome. Critical care medicine. 2006;34(12):2946-51.

5. Tobin AE, Santamaria JD. After-hours discharges from intensive care are associated with increased mortality. The Medical journal of Australia. 2006;184(7):334-7.

6. Pilcher DV, Duke GJ, George C, Bailey MJ, Hart G. After-hours discharge from intensive care increases the risk of readmission and death. Anaesthesia and intensive care. 2007;35(4):477-85.

7. Laupland KB, Shahpori R, Kirkpatrick AW, Stelfox HT. Hospital mortality among adults admitted to and discharged from intensive care on weekends and evenings. Journal of critical care. 2008;23(3):317-24.

8. Singh MY, Nayyar V, Clark PT, Kim C. Does after-hours discharge of ICU patients influence outcome? Critical care and resuscitation : journal of the Australasian Academy of Critical Care Medicine. 2010;12(3):156-61.

9. Uusaro A, Kari A, Ruokonen E. The effects of ICU admission and discharge times on mortality in Finland. Intensive care medicine. 2003;29(12):2144-8.

10. Hanane T, Keegan MT, Seferian EG, Gajic O, Afessa B. The association between nighttime transfer from the intensive care unit and patient outcome. Critical care medicine. 2008;36(8):2232-7.

11 Santamaria JD, Duke GJ, Pilcher DV, Cooper DJ, Moran J, Bellomo R. The timing of discharge from the intensive care unit and subsequent mortality. A prospective, multicenter study. American journal of respiratory and critical care medicine. 2015;191(9):1033-9.

12. Gantner D, Farley KJ, Bailey M, Huckson S, Hicks P, Pilcher D. Mortality related to after-hours discharge from intensive care in Australia and New Zealand, 2005–2012. Intensive care medicine. 2014;40(10):1528-35.

13. Azevedo LC, de Souza IA, Zygun DA, Stelfox HT, Bagshaw SM. Association Between Nighttime Discharge from the Intensive Care Unit and Hospital Mortality: A Multi-Center Retrospective Cohort Study. BMC health services research. 2015;15:378.

14. Laupland KB, Misset B, Souweine B, Tabah A, Azoulay E, Goldgran-Toledano D, et al. Mortality associated with timing of admission to and discharge from ICU: a retrospective cohort study. Ibid. 2011;11:321.

15 Hosein FS, Roberts DJ, Turin TC, Zygun D, Ghali WA, Stelfox HT. A meta-analysis to derive literature-based benchmarks for readmission and hospital mortality after patient discharge from intensive care. Critical care (London, England). 2014;18(6):715.

16. Elliott M, Bn L, Worrall-Carter, Page K. Factors associated with in-hospital mortality following ICU discharge: a comprehensive review. Carter. 2012;22:120-5.


**Table S2.** **The justifications for the study exclusions (n=26).**

| **Studies Not include** | **Reasons (According to PICOS)** |
| --- | --- |
| Moreira E.,2016 [1] | This is a meeting abstract, without full text. We cannot obtain the adjusted effect size and its 95% CI with adjustment for the severity of the disease after contacting authors. |
| Perry J.,2016 [2] | This is a meeting abstract, without full text. It is not a cohort study. |
| Bramma YL ,2012 [3] | This is a meeting abstract, without full text. We cannot obtain the adjusted effect size and its 95% CI with adjustment for the severity of the disease after contacting authors. |
| Edie S,2015 [4] | This is a meeting abstract, without full text. We cannot obtain the adjusted effect size and its 95% CI with adjustment for the severity of the disease after contacting authors. |
| Ahmed M,2015 [5] | This is a meeting abstract, without full text. And the target end point is not mortality. |
| Gopal S, 2010 [6] | This is a meeting abstract, without full text. And the target end point is not mortality. |
| Harvey S,2005 [7] | This is a meeting abstract, without full text. We cannot obtain sufficient information to perform quality assessment after contacting authors. |
| Santamaria JD,2011 [8] | This is a meeting abstract, the full text was published in 2015(Santamaria JD ,2015 [27] ) |
| Santamaria JD,2007[9] | This is an Editorial without relevant data reported. |
| Brasel K ,2008[10] | This is an Editorial without relevant data reported. |
| Morris PE, 2009[11] | This is an Editorial without relevant data reported. |
| Halpern SD , 2015[12] | This is an Editorial without relevant data reported. |
| Dorrance M , 2015[13] | It is not a cohort study. |
| Munro PA,2014[14] | It is not a cohort study. |
| Garland A,2013[15] | Not relevant, not the target exposure: discharge delay. |
| Ouanes I,2012[16] | It is not a cohort study. |
| Santamaria JD,2011[17] | This is a meeting abstract, without full text. And the target end point is not mortality. |
| Obel N,2007[18] | This is a cohort study, it explores the relationship between discharge from an intensive care unit on weekends and hospital mortality, but it reports the OR and its 95% CI with not adjustment for the severity of the disease. |
| Bristlow PJ,2003 [19] | This is a meeting abstract, without full text. We cannot obtain the adjusted effect size and its 95% CI with adjustment for the severity of the disease after contacting authors. |
| Karnik A,2001[20] | This is a meeting abstract, without full text. We cannot obtain the adjusted effect size and its 95% CI with adjustment for the severity of the disease after contacting authors. |
| O'Keeffe J, 2016[21 | Not relevant, not the target exposure: delayed discharges. |
| Churpek MM,2016[22] | Not relevant, not the target exposure: delayed intensive care unit (ICU) transfer. |
| Whitaker M, 2015[23] | Not relevant, not the target exposure: delayed ICU discharges. |
| Vollam SA,2015[24] | This is a systematic review protocol without relevant data reported. |
| Churpek MM,2013[25] | Not relevant, not the target exposure: delayed ICU discharges. |
| Chrusch CA,2009[26] | Not relevant, not the target exposure: patient occupancy. |
|  |  |
|  |  |

**Reference**

1. Moreira E, Verga F, Barbato M, Burghi G. Effects of ICU weekend admission and discharge on mortality. Critical Care. 2016;20.

2. Perry J, Hines H. ‘sooner rather than later’: how delayed discharge from critical care leads to increased out of hours discharges and subsequent increase in in-hospital mortality. Crit Care 2016 20(Suppl 2): P395

3. Bramma YL, Allan R, Sundaram R. Out-of-hours discharge from the ICU: Defining the out-of-hours period and its effect on mortality. Critical Care. 2012;16:S182.

4. Edie S, Burt K, Paddle J. Association between out-of-hours discharge and mortality in adult patients leaving critical care. Critical Care. 2015;19:S196.

5. Ahmed M. Lumley G, Nourse S. et al. Out-of-hours discharge from critical care: does it matter? Crit Care (2015) 19: P526. doi:10.1186/cc14606

## 6. Gopal S, Terry L, Corbett C. Association between out of hours discharge from the ICU and subsequent readmission. Crit Care (2010) 14: P476. doi:10.1186/cc8708

7. Harvey, S., Wunsch, H., Welch, C., Harrison D., Rowan K. Hospital mortality associated with day and time of discharge from intensive care units in the United Kingdom. Crit Care (2005) 9: P238. doi:10.1186/cc3301

8. Santamaria JD, Duke G, Pilcher D, Cooper J. After-hours discharge from ICU is a risk factor for in-hospital mortality in a large multi-center prospective study. American journal of respiratory and critical care medicine. 2011;183(1).

9 .Santamaria J. After-hours discharge from intensive care: Impact on outcome. Anaesthesia and Intensive Care. 2007;35(4):475-6.

10. Brasel K. Can we safely discharge patients from the intensive care unit after hours? Critical care medicine. 2008;36(8):2443-4.

11. Morris PE. Assessing ICU transfers at night: a call to reduce mortality and readmission risk. Am J Crit Care. 2009;18(1):6-8.

12. Halpern SD. Nighttime in the intensive care unit. A lens into the value of critical care delivery. Am J Respir Crit Care Med. 2015;191(9):974-5.

13. Dorrance M, Broughton N. ICU discharge time-meeting the standard but missing the point? Anaesthesia. 2015;70:10.

14. Munro PA, Keane F. In-hospital mortality following intensive care unit discharge: An audit of adult patients in a district general hospital. Journal of the Intensive Care Society. 2014;15(1):S61.

15. Garland A, Connors AF, Jr. Optimal timing of transfer out of the intensive care unit. American journal of critical care : an official publication, American Association of Critical-Care Nurses. 2013;22(5):390-7.

16. Ouanes I, Schwebel C, Français A, Bruel C, Philippart F, Vesin A, et al. A model to predict short-term death or readmission after intensive care unit discharge. Journal of critical care. 2012;27(4):422.e1-.e9.

17. Santamaria JD, Duke G, Pilcher D, Cooper J. A prospective multi-center study of discharge from intensive care. Factors associated with readmission. American journal of respiratory and critical care medicine. 2011;183(1).

18**.** Obel N, Schierbeck J, Pedersen L, Storgaard M, Pedersen C, Sørensen HT, et al. Mortality after discharge from the intensive care unit during the early weekend period: A population-based cohort study in Denmark. Acta anaesthesiologica Scandinavica. 2007;51(9):1225-30.

## 19. Bristlow PJ. Discharge out of hours are associated with increased mortality. Anaesthesia Intensive Care 2003;31:222.

20. Karnik A, Fraser J, Cook D. Out-of-hours discharge: are we risking our patients’ lives? Anaesthesia Intensive Care. 2001;29(2):200

21**.** O'Keeffe J, O'Dea J, O'Brien F. Delayed admissions and discharges from the intensive care unit, the reasons, and the impact on patients. Irish Journal of Medical Science. 2016;185(2):S107.

22**.** Churpek MM, Wendlandt B, Zadravecz FJ, Adhikari R, Winslow C, Edelson DP. Association between intensive care unit transfer delay and hospital mortality: A multicenter investigation. Journal of hospital medicine. 2016.

23**.** Whitaker M, Spivey MH. Delayed ICU discharges and medical follow-up: A cause of increased mortality? Critical Care. 2015;19:S179.

24**.** Vollam SA, Dutton SJ, Young D, Watkinson PJ. Out-of-hours discharge from intensive care, in-hospital mortality and intensive care readmission rates: a systematic review protocol. Systematic reviews. 2015;4:93.

25.Churpek MM, Yuen TC, Edelson DP. Delayed intensive care unit transfer is associated with increased mortality in ward patients. American journal of respiratory and critical care medicine. 2013;187.

26**.** Chrusch CA, Olafson KP, McMillan PM, Roberts DE, Gray PR. High occupancy increases the risk of early death or readmission after transfer from intensive care. Critical care medicine. 2009;37(10):2753-8.

27. Santamaria JD, Duke GJ, Pilcher DV, Cooper DJ, Moran J, Bellomo R. The timing of discharge from the intensive care unit and subsequent mortality. A prospective, multicenter study. American journal of respiratory and critical care medicine. 2015;191(9):1033-9.

**Table S3.** **Newcastle-Ottawa quality assessment of included studies.**

| **Study** | **Selection** | | | | **Comparability** | | **Outcome** | | | **Total score** |
| --- | --- | --- | --- | --- | --- | --- | --- | --- | --- | --- |
|  | *1)* | *2)* | *3)* | *4)* | *1a)* | *1b)* | *1)* | *2)* | *3)* |  |
| Santamaria et al. /2015 | adult patients discharged alive from the ICU at night (somewhat representative)/ one star | drawn from same population as exposed cohort / one star | medical records/ one star | yes/ one star | yes/ one star | yes/ one star | medical records/ 0 star | yes (2009.9- 2010.2) / one star | all patients with complete follow up/ one star | 8 |
| Azevedo et al. /2015 | adult patients discharged alive from the ICU at night (somewhat representative)/ one star | drawn from same population as exposed cohort / one star | medical records, database/ one star | yes/ one star | yes/ one star | no /0 star | medical records, database/ 0 star | yes (2002.7 -2009 .12) / one star | unreported/ 0 star | 6 |
| Gantner et al  ./2014 | adult patients discharged alive from the ICU at night (somewhat representative)/ one star | drawn from same population as exposed cohort / one star | medical records, database/ one star | yes / one star | yes/ one star | yes/ one star | medical records, database/0 star | yes (2005.1 -2012.12) / one star | unreported/ 0 star | 7 |
| Laupland et al. /2011 | adult patients discharged alive from the ICU at night (somewhat representative)/ one star | drawn from same population as exposed cohort / one star | medical records, database/ one star | yes/ one star | yes/ one star | yes/ one star | medical records, database/ 0 star | yes (2006.1 -2010.11) / one star | unreported/ 0 star | 7 |
| Singh et al.  /2010 | adult patients discharged alive from the ICU at night (somewhat representative)/ one star | drawn from same population as exposed cohort / one star | medical records, database/ one star | yes/ one star | yes/ one star | no /0 star | medical records, database/ 0 star | yes(2004.1.1-2006.12.31) / one star | unreported/ 0 star | 6 |
| Hanane et al.  /2008 | adult patients discharged alive from the ICU at night (somewhat representative)/ one star | drawn from same population as exposed cohort / one star | medical records, database/ one star | yes/ one star | yes/ one star | yes/ one star | medical records, database/ 0 star | yes(2003-2006) / one star | unreported/ 0 star | 7 |
| Laupland et al. /2008 | adult patients discharged alive from the ICU at night (somewhat representative)/ one star | drawn from same population as exposed cohort / one star | medical records, database/ one star | yes/ one star | yes/ one star | no /0 star | medical records, database/ 0 star | yes(2000.1.1-2006.12.31) / one star | unreported/ 0 star | 6 |
| Pilcher et al.  /2007 | adult patients discharged alive from the ICU at night (somewhat representative)/ one star | drawn from same population as exposed cohort / one star | medical records, database/ one star | yes/ one star | yes/ one star | no /0 star | medical records, database/ 0 star | yes(2003.1.1-2004.12.31) / one star | unreported/ 0 star | 6 |
| Tobin et al.  /2006 | adult patients discharged alive from the ICU at night (somewhat representative)/ one star | drawn from same population as exposed cohort / one star | medical records, database/ one star | yes/ one star | yes/ one star | no /0 star | medical records, database/ 0 star | yes(1992.1.1-2002.12.31) / one star | unreported/ 0 star | 6 |
| Priestap et al.  /2006 | adult patients discharged alive from the ICU at night (somewhat representative)/ one star | drawn from same population as exposed cohort / one star | medical records, database/ one star | yes/ one star | yes/ one star | no /0 star | medical records, database/ 0 star | yes (2001.1 -2004.9) / one star | unreported/ 0 star | 6 |
| Duke et al.  /2004 | adult patients discharged alive from the ICU at night (somewhat representative)/ one star | drawn from same population as exposed cohort / one star | medical records, database/ one star | yes/ one star | yes/ one star | yes/ one star | medical records, database/ 0 star | yes (1999.1.1-2003.4.30) / one star | unreported/ 0 star | 7 |
| Uusaro et al.  /2003 | adult patients discharged alive from the ICU at night (somewhat representative)/ one star | drawn from same population as exposed cohort / one star | medical records, database/ one star | yes/ one star | yes/ one star | yes/ one star | medical records, database/0 star | yes (1998.1–2001.6) / one star | unreported/ 0 star | 7 |
| Beck et al.  /2002 | adult patients discharged alive from the ICU at night (somewhat representative)/ one star | drawn from same population as exposed cohort / one star | medical records, database/ one star | yes/ one star | yes/ one star | no /0 star | medical records, database/0 star | yes (1996.1.1 -2000 .3.31) / one star | unreported/ 0 star | 6 |
| Goldfrad et al.  /2000 | adult patients discharged alive from the ICU at night (somewhat representative)/ one star | drawn from same population as exposed cohort / one star | medical records, database/ one star | yes/ one star | yes/ one star | no /0 star | medical records, database/ 0 star | yes (1995-1998) / one star | unreported/ 0 star | 6 |
| **Selection:** 1) Representativeness of the exposed cohort; 2) Selection of the non-exposed cohort; 3) Ascertainment of exposure; 4) Demonstration that outcome of interest was not present at start of study; | | | | | | | | | | |
|  |  |  |  |  |  |  |  |  |  |  |
| **Comparability:** 1a) study controls for (most important factor--- **severity of illness on ICU admission or discharge**); 1b) study controls for any additional factor(**treatment limitation orders** ); | | | | | | | | | | |
| **Outcome:** 1) Assessment of outcome; 2) Was follow-up long enough for outcomes to occur (≥6 month); 3) Adequacy of follow up of cohorts (≥80%). | | | | | | | | | | |
