# Supplementary figures and images for: Association between time of discharge from ICU and hospital mortality: a systematic review and meta-analysis
Source: Crit Care. 2016 Dec 1;20:390. doi: 10.1186/s13054-016-1569-x (PMC5131545; doi:10.1186/s13054-016-1569-x)

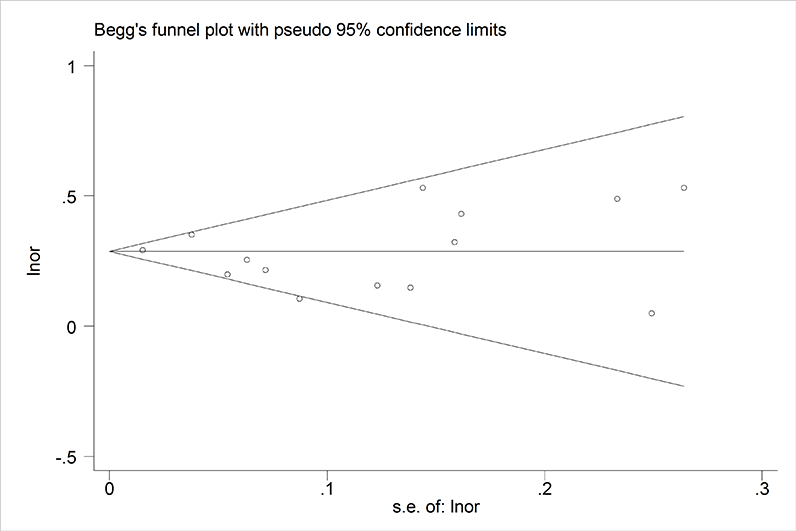

Supplement: Additional file 3: Figure S1. — Forest plots of the association between nighttime discharge from the ICU and hospital mortality stratified by geographic region. The size of each square is proportional to the study weight. Open diamonds represent the pooled OR. D + L refers to random effects and I-V to fixed effects. Figure S2. Forest plots of the association between nighttime discharge from the ICU and hospital mortality stratified by study design. The size of each square is proportional to the study weight. Open diamonds represent the pooled OR. D + L refers to random effects and I-V to fixed effects. Figure S3. Forest plots of the association between nighttime discharge from the ICU and hospital mortality stratified by the total discharge number. The size of each square is proportional to the study weight. Open diamonds represent the pooled OR. D + L refers to random effects and I-V to fixed effects. Figure S4. Funnel plots showing the association of nighttime discharge from the ICU with hospital mortality. s.e. refers to standard error, or refers to odds ratio. Figure S5. Funnel plots showing the association of weekend discharge from the ICU with hospital mortality. s.e. refers to standard error, or refers to odds ratio. (ZIP 395 kb) [file 13054_2016_1569_MOESM3_ESM.zip › Figure S4.tif]

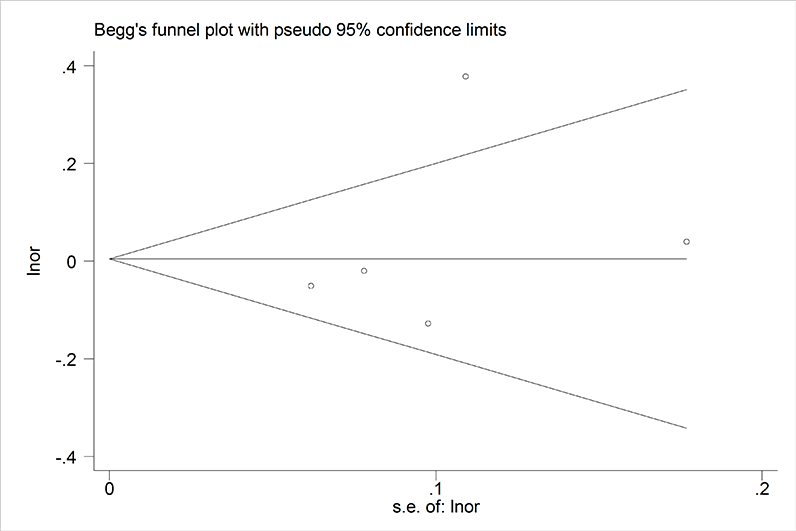

Supplement: Additional file 3: Figure S1. — Forest plots of the association between nighttime discharge from the ICU and hospital mortality stratified by geographic region. The size of each square is proportional to the study weight. Open diamonds represent the pooled OR. D + L refers to random effects and I-V to fixed effects. Figure S2. Forest plots of the association between nighttime discharge from the ICU and hospital mortality stratified by study design. The size of each square is proportional to the study weight. Open diamonds represent the pooled OR. D + L refers to random effects and I-V to fixed effects. Figure S3. Forest plots of the association between nighttime discharge from the ICU and hospital mortality stratified by the total discharge number. The size of each square is proportional to the study weight. Open diamonds represent the pooled OR. D + L refers to random effects and I-V to fixed effects. Figure S4. Funnel plots showing the association of nighttime discharge from the ICU with hospital mortality. s.e. refers to standard error, or refers to odds ratio. Figure S5. Funnel plots showing the association of weekend discharge from the ICU with hospital mortality. s.e. refers to standard error, or refers to odds ratio. (ZIP 395 kb) [file 13054_2016_1569_MOESM3_ESM.zip › Figure S5.tif]

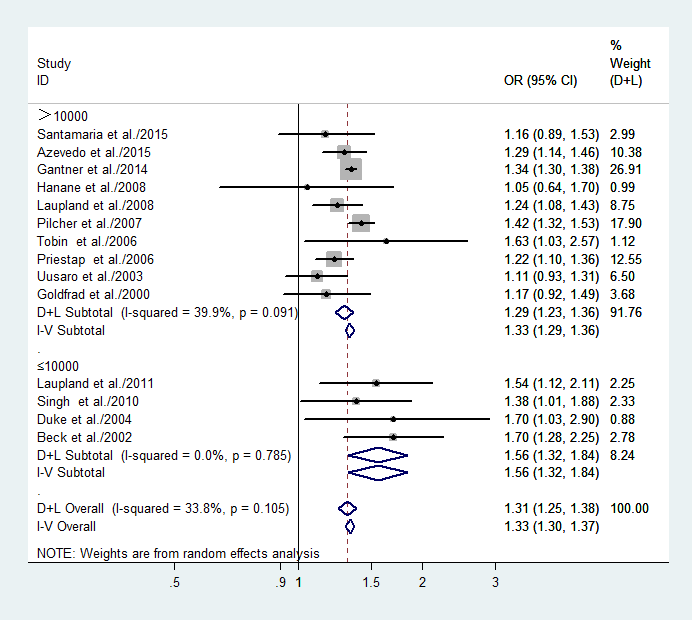

Supplement: Additional file 3: Figure S1. — Forest plots of the association between nighttime discharge from the ICU and hospital mortality stratified by geographic region. The size of each square is proportional to the study weight. Open diamonds represent the pooled OR. D + L refers to random effects and I-V to fixed effects. Figure S2. Forest plots of the association between nighttime discharge from the ICU and hospital mortality stratified by study design. The size of each square is proportional to the study weight. Open diamonds represent the pooled OR. D + L refers to random effects and I-V to fixed effects. Figure S3. Forest plots of the association between nighttime discharge from the ICU and hospital mortality stratified by the total discharge number. The size of each square is proportional to the study weight. Open diamonds represent the pooled OR. D + L refers to random effects and I-V to fixed effects. Figure S4. Funnel plots showing the association of nighttime discharge from the ICU with hospital mortality. s.e. refers to standard error, or refers to odds ratio. Figure S5. Funnel plots showing the association of weekend discharge from the ICU with hospital mortality. s.e. refers to standard error, or refers to odds ratio. (ZIP 395 kb) [file 13054_2016_1569_MOESM3_ESM.zip › Figure S3.tif]

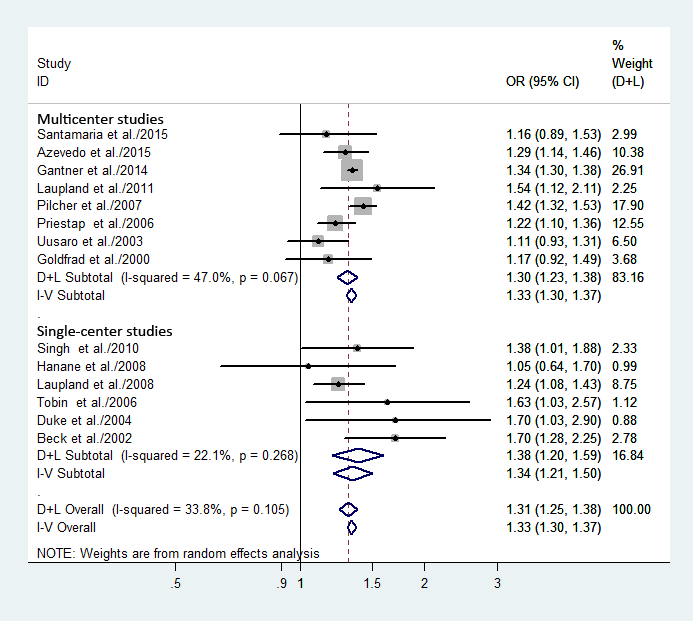

Supplement: Additional file 3: Figure S1. — Forest plots of the association between nighttime discharge from the ICU and hospital mortality stratified by geographic region. The size of each square is proportional to the study weight. Open diamonds represent the pooled OR. D + L refers to random effects and I-V to fixed effects. Figure S2. Forest plots of the association between nighttime discharge from the ICU and hospital mortality stratified by study design. The size of each square is proportional to the study weight. Open diamonds represent the pooled OR. D + L refers to random effects and I-V to fixed effects. Figure S3. Forest plots of the association between nighttime discharge from the ICU and hospital mortality stratified by the total discharge number. The size of each square is proportional to the study weight. Open diamonds represent the pooled OR. D + L refers to random effects and I-V to fixed effects. Figure S4. Funnel plots showing the association of nighttime discharge from the ICU with hospital mortality. s.e. refers to standard error, or refers to odds ratio. Figure S5. Funnel plots showing the association of weekend discharge from the ICU with hospital mortality. s.e. refers to standard error, or refers to odds ratio. (ZIP 395 kb) [file 13054_2016_1569_MOESM3_ESM.zip › Figure S2.tif]

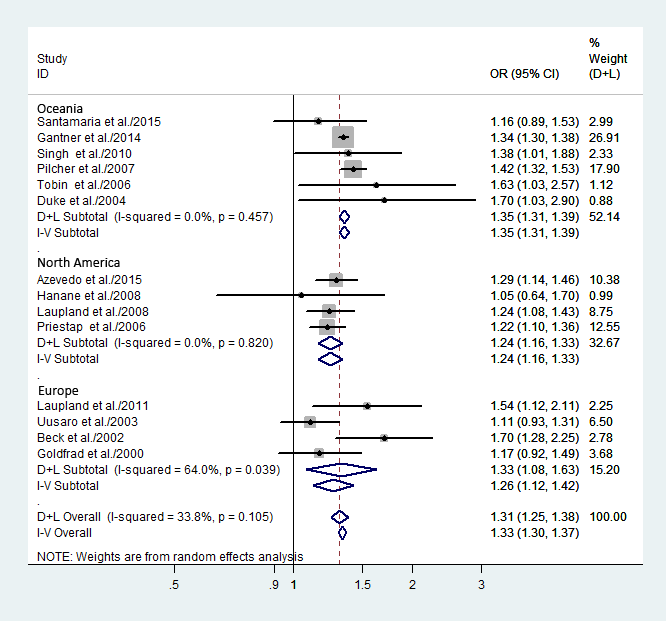

Supplement: Additional file 3: Figure S1. — Forest plots of the association between nighttime discharge from the ICU and hospital mortality stratified by geographic region. The size of each square is proportional to the study weight. Open diamonds represent the pooled OR. D + L refers to random effects and I-V to fixed effects. Figure S2. Forest plots of the association between nighttime discharge from the ICU and hospital mortality stratified by study design. The size of each square is proportional to the study weight. Open diamonds represent the pooled OR. D + L refers to random effects and I-V to fixed effects. Figure S3. Forest plots of the association between nighttime discharge from the ICU and hospital mortality stratified by the total discharge number. The size of each square is proportional to the study weight. Open diamonds represent the pooled OR. D + L refers to random effects and I-V to fixed effects. Figure S4. Funnel plots showing the association of nighttime discharge from the ICU with hospital mortality. s.e. refers to standard error, or refers to odds ratio. Figure S5. Funnel plots showing the association of weekend discharge from the ICU with hospital mortality. s.e. refers to standard error, or refers to odds ratio. (ZIP 395 kb) [file 13054_2016_1569_MOESM3_ESM.zip › Figure S1.tif]
